# Optic neuropathy in craniosynostosis

**DOI:** 10.3389/fopht.2023.1303723

**Published:** 2024-01-10

**Authors:** Tais Estrela, Linda R. Dagi

**Affiliations:** Department of Ophthalmology, Boston Children’s Hospital, Harvard Medical School, Boston, MA, United States

**Keywords:** craniosynostosis, optic neuropathy, papilledema, optic atrophy, optical coherence tomography

## Abstract

Craniosynostosis (CS) or the premature fusion of one or more cranial sutures *in utero*, or during the first years of life, can present in isolation or as a multisystem clinical disorder with a particular impact on visual function. Among ophthalmic complications, optic neuropathy is a significant cause of irreversible vision loss in these patients. Children with CS are at higher risk of developing elevated intracranial pressure which can lead to papilledema and, ultimately, optic atrophy. In addition, sometimes associated obstructive sleep apnea, abnormalities in central nervous system venous development, and Chiari malformation may contribute to optic neuropathy. Ophthalmologists have an important role in managing a number of coexistent ophthalmologic complications such as strabismus, anisometropia, amblyopia, ptosis, and exposure keratopathy in addition to maintaining surveillance for early signs of optic neuropathy; they play a critical consultative role contributing to the decision for primary or repeat decompressive surgery. In this article, we aim to review the etiology, diagnostic approach, and management of optic neuropathies in patients with craniosynostosis.

## 1 Introduction

Craniosynostosis (CS) is the premature fusion of one or more cranial sutures during fetal development and childhood and occurs in 2100-2500 births (1, 2). Among systemic complications, visual impairment is an important feature of this condition and can be caused by multiple mechanisms such as strabismus, refractive error, amblyopia, corneal scarring from exposure keratopathy (3), and, importantly, optic neuropathy, which has been reported as a prevalent cause of irreversible vision loss in these patients (4, 5). In a review of 141 children with cranio-synostotic syndromes of Apert, Crouzon, Pfeiffer, and Saethre–Chotzen Khan et al. (5) found that approximately 40% had visual acuity 6/12 or worse in the better eye. Tay et al. (6) reported a prevalence of visual impairment of 33.5% in patients with craniosynostotic syndromes of Apert, Crouzon, Pfeiffer, Saethre–Chotzen and craniofrontonasal dysplasia and found optic atrophy in 16.7% of patients.

Given the high prevalence of visual impairment and the risk of optic neuropathy, ophthalmologists have the challenging role of detecting early signs of optic nerve damage to guide treatment and prevent irreversible vision loss. This review focuses on the cause, diagnostic methods, and management of optic neuropathy in patients with craniosynostosis.

## 2 Etiology

Elevated intracranial pressure (ICP) is a common complication of CS and a known cause of papilledema that, if untreated, causes irreversible retinal neuroaxonal damage leading to optic atrophy. The risk of elevated ICP varies from 15-20% in non-syndromic craniosynostosis and 30-40% in syndromic cases (3, 7, 8). Patients with multi-sutural involvement are at higher risk of elevated ICP and papilledema. Greene et al. (7) found that 76.9% of children with phenotypically unusual combined craniosynostoses had clinical or radiographic signs of increased ICP. In patients with non-syndromic suture synostosis, the ones with midline suture synostosis (sagittal or metopic) seem to have a higher risk for the development of elevated ICP (9). Importantly, the prevalence of optic neuropathy may differ among the CS syndromes. In a recent study, Fearon et al. (10) evaluated the prevalence of optic atrophy in 253 patients with CS followed for an average period of 10 years. They did not find optic atrophy in patients with either Saethre-Chotzen or Muenke syndrome and found a statistically significant lower prevalence noted among those with Apert syndrome (7.8%) compared to those with Crouzon (27.9%) and Pfeiffer syndromes (23.1%), P<0.001.

Although it is intuitive to think that raised ICP is secondary to the mismatch between cerebral growth and cranial vault volume, there is a body of evidence suggesting that this is not the only cause (11). It has been shown that patients with Apert syndrome, for example, tend to have greater-than-normal intracranial volume (12). Marucci et al. (13) evaluated the natural history of raised ICP in these patients. In their cohort of 24 children with Apert syndrome, two required shunt procedures for hydrocephalus, and two had their raised ICP treated by correcting upper airway obstruction alone. Their results support prior authors who suggested that other mechanisms, such as obstructive or impaired cerebrospinal fluid (CSF) absorption, sleep apnea, and anomalous venous drainage, are involved in the pathogenesis of raised ICP in patients with CS (14–16). Importantly, these mechanisms can coexist, leading to worse visual outcomes. Nguyen et al. (17) found that patients with a history of obstructive sleep apnea, in addition to recurrent elevated ICP, had a greater risk of optic neuropathy.

In addition to chronic elevation of ICP, optic nerve canal anomalies may possibly contribute to optic nerve damage in CS. Hariri et al. (18) showed that anatomic parameters of the optic nerve canal for syndromic CS patients were statistically narrower than those of healthy children and raised the hypothesis that the anatomic findings could be involved in the physiopathology of optic neuropathy in children with CS. However, the association between optic canal parameters and optic neuropathy has not been demonstrated to date.

Of importance, prior papilledema and anatomic changes of the optic nerve canal have been implicated in axoplasmic stasis, which can lead to the development of peripapillary hyperreflective ovoid mass-like structures (PHOMS) (19–21). PHOMS appear to result from herniated optic nerve fibers bulging into the peripapillary region, causing elevation of the RNFL and, therefore, pseudopapilledema (21). Although, at this point, there is no published data regarding the prevalence and significance of PHOMS in patients with CS, the presence of PHOMS may be a confounding factor in assessing papilledema in CS (18).

## 3 Diagnostic testing

Ophthalmologic assessment of patients with CS is extremely important and can guide surgical management and help to avoid invasive ICP measurement tests.

However, vision testing is frequently affected by concomitant ocular diseases such as exposure keratopathy and amblyopia (5). In addition, visual acuity and visual field testing can be challenging in the subset of this population with impaired cognition. In very young or pre-verbal children, visual acuity testing with Teller acuity cards, color vision testing by tracing color plates, and confrontation visual field testing provide useful information. Haredy et al. (22) suggest the use of pattern reversal visually-evoked potentials (pVEP), a test that does not require cooperation, to detect visual pathway dysfunction in patients with CS. Although, it has been shown that there is a high prevalence of abnormal pVEP in patients with CS (23), the test is not sensitive to small changes visual in function.

Historically, fundoscopy was the primary objective test in the evaluation of the optic nerve. Although the presence of optic nerve swelling can indicate elevated ICP, it is not always a sufficiently sensitive screening tool (8, 24). This is especially true in cases where optic atrophy has already occurred, or in cases where the presence of optic disc drusen (ODD) or PHOMS are present, causing optic nerve head elevation.

Ocular ultrasound is also a non-invasive test that can be helpful in the assessment of papilledema in young children, and children with corneal opacities, and can also be helpful in differentiating true papilledema from pseudo papilledema (25, 26). Farazdaghi et al. (27) found that optic nerve sheath widening of >4.5mm had a modest positive predictive value for the presence of papilledema (65%, 95% CI: 45–80%); however, optic nerve sheath diameter of ≤ 4.5 mm had a 96% predictive value that papilledema was not present. The downside is that this technology is limited in providing quantitative information on optic nerve structure, and does not reliably indicate worsening optic neuropathy in the presence of pre-existing optic atrophy. In addition, atypical orbital volume and shape may impact specificity for this parameter, particularly in patients with craniosynostosis. Similarly, magnetic resonance imaging (MRI) and computed tomography (CT) can reveal signs of papilledema and optic atrophy but offer limited data on nerve structure. Importantly, however, these radiologic tests provide anatomic information and help to identify the cause of optic nerve damage.

Advances in ophthalmic imaging with spectral-domain optical coherence tomography (SD-OCT) and the development of faster scans have enabled widespread use of OCT in the pediatric population. OCT can provide quantitative structural information and allows for the identification of small changes in the optic nerve structure, significantly supporting the diagnosis and follow-up of papilledema and optic atrophy in children with CS. Dagi et al. (28) reported that peripapillary retinal nerve fiber layer (RNFL) thickness measurements by SD-OCT were 88% sensitive at detecting optic atrophy and 60% sensitive in detecting papilledema in patients with CS. More recently, Swanson et al. (29) found a statistically significant correlation between RNFL thickness and elevated ICP. In addition to RNFL, newer modalities such swept-source (SS) OCT and enhanced depth imaging (EDI) SD-OCT provide better visualization of the optic nerve head anatomy aiding identification of drusen and PHOMS, to help distinguish papilledema from pseudo papilledema (30).

Den Ottelander et al. (31) further explored OCT in children with CS. They looked at the prevalence of retinal thickness abnormalities, including peripapillary and macula retinal thickness, in syndromic and complex craniosynostosis, and evaluated its correlation with visual acuity. They found that the decrease in the macular ganglion cell layer- inner plexiform layer (GCL-IPL) thickness was significantly associated with a worse VA, but there was no significant correlation between RNFL and VA. Their results suggested that a macular scan provides more information relevant to visual function than RNFL. Most recently, Chang et al. (32) reported a significant association between visual acuity and lower ganglion cell layer (GCL) volume. In addition, the authors demonstrated that macula GCL volume<1.02 mm^3^ had a sensitivity of 83% and specificity of 77% in identifying optic atrophy; they also demonstrated that the presence of obstructive sleep apnea was an independent risk factor for the development optic atrophy in a cohort of 58 patients with CS (32).

Although promising, the use of OCT in patients with CS has limitations. The exam requires cooperation, which can be challenging for young children, and the presence of nystagmus in some cases limits the acquisition of quality data.

## 4 Management

The primary management of optic neuropathy in CS is to treat elevated intracranial pressure to prevent optic nerve damage. Primary craniofacial surgical intervention is usually performed in the first months or years of life and typically involves opening fused sutures endoscopically or expanding the cranial vault via a variety of other procedures that permit more normal cerebral growth and neurological development; sometimes endoscopic third ventriculostomy is used to augment ICP control. Surgical techniques are mainly divided between open vault cranioplasty and strip craniectomy, which can be endoscopically or open. Mackinnon et al. (33) specifically compared ophthalmologic outcomes and found that children with unicoronal synostosis treated by early endoscopic strip craniectomy developed less severe V-pattern strabismus, required fewer surgical procedures to treat this strabismus and had less aniso-astigmatism than those treated by later frontal-orbital advancement. More recently, these outcomes were confirmed by Elhusseiny et al (34) in a cohort of 120 infants with unicoronal synostosis treated with either early endoscopic strip craniectomy or later open cranial vault expansion. Dohlman et al. (35) found that early endoscopic strip craniectomy of multiple fused sutures in patients with Apert syndrome resulted in more normalized eyelid anatomy, less rectus muscle excyclorotation and less severe V-pattern strabismus than that seen in patients with Apert syndrome primarily treated with open cranial vault expansion; importantly, they also showed that sustained intracranial pressure control appeared possibly less optimal, though not statistically different, in the endoscopic versus the open-vault treated cohort.

Of note, surgical management of optic neuropathy in CS is not limited to correcting cranial abnormalities. Procedures such as ventriculoperitoneal shunt and endoscopic third ventriculostomy may be necessary to address progressive hydrocephalus (36), and surgery to correct airway obstruction is critical since it has been demonstrated that obstructive sleep apnea is a major risk factor for the development of optic atrophy (32, 37).

Regardless of the surgical procedure, recurrence of elevated ICP is not uncommon (38), and a multidisciplinary approach that includes scheduled periodic ophthalmologic follow-up is necessary to evaluate for signs of recurrence of papilledema or progression to optic nerve atrophy. Best corrected visual acuity (optotype or Teller), serial OCT measurements if possible, assessment and treatment of associated obstructive sleep apnea if present, and consideration of interval neuroimaging if history, signs, or symptoms suggest a need are important considerations. Ophthalmologists plan a critical advisory role for the craniofacial and neurosurgical team, often guiding when surgical intervention is necessary to prevent vision loss.

## 5 Conclusion

In summary, this review illustrates the importance of close ophthalmologic follow-up to prevent optic neuropathy in patients with CS and the utility of objective metrics for early detection of optic nerve damage. Although advances in OCT have added critical structural information about the optic nerve in patients with CS, there are still important questions to answer. Future research is needed to explore longitudinal rates of RNFL change, to identify patients with CS at higher risk of developing optic atrophy, and to evaluate the correlation of OCT macula parameters, and possibly OCT angiography to optimize surveillance and management of this population.

## Author contributions

TE: Writing – review & editing. LD: Supervision, Writing – review & editing.
